# Antibiotikaeinsatz zu Prophylaxe und empirischer Therapie von frakturassoziierten Infektionen in Deutschland: Eine Umfrage an 44 Kliniken

**DOI:** 10.1007/s00113-022-01200-0

**Published:** 2022-06-24

**Authors:** Susanne Bärtl, Nike Walter, Siegmund Lang, Florian Hitzenbichler, Markus Rupp, Volker Alt

**Affiliations:** 1 Klinik und Poliklinik für Unfallchirurgie, Universitätsklinikum Regensburg, Franz-Josef-Strauß-Allee 11, 93053 Regensburg, Deutschland; 2 Abteilung für Krankenhaushygiene und Infektiologie, Universitätsklinikum Regensburg, Franz-Josef-Strauß-Allee 11, 93053 Regensburg, Deutschland

**Keywords:** Antibiotikaprophylaxe, Empirische Antibiotikatherapie, Lokale Antibiotikatherapie, Implantatassoziierte Infektion, Fraktur, Antibiotic prophylaxis, Empirical antibiotic therapy, Local antibiotic therapy, Implant-associated infection, Fracture

## Abstract

**Hintergrund:**

Antibiotika (AB) spielen eine wichtige Rolle in der Prophylaxe und Behandlung von Infektionen in der Unfallchirurgie. Dennoch scheint es gerade bei der Infektionsprophylaxe nach offenen Frakturen und auch bei der empirischen Therapie von frakturassoziierten Infektionen (FRI) große Unterschiede zwischen einzelnen Kliniken zu geben.

**Methodik:**

An deutschen Universitäts- und berufsgenossenschaftlichen Kliniken wurde eine Umfrage zu Prophylaxe und empirischer AB-Therapie von FRI durchgeführt. Die AB-Regime wurden mit dem Resistenzprofil der Erreger bei 86 FRI-Patienten verglichen, um die theoretische Wirksamkeit der jeweiligen Therapien zu ermitteln.

**Ergebnisse:**

Von 71 Kliniken antworteten insgesamt 44 (62,0 %). Bei geschlossenen Frakturen zeigte sich mit der Verwendung von Cephalosporinen in 95,5 % der Kliniken ein einheitliches Bild. Für offene Frakturen wurden 8 verschiedene AB-Regime berichtet, wobei Aminopenicilline/β-Lactamase-Inhibitor (BLI) (31,8 %) am häufigsten genannt wurden. Für die empirische Therapie der FRI wurden 12 verschiedene AB-Regime angegeben, am häufigsten Aminopenicilline/BLI (31,8 %), Cephalosporine (31,8 %) und Ampicillin/Sulbactam + Vancomycin (9,1 %). Hinsichtlich der empirischen Therapie der FRI zeigten sich niedrige Sensibilitätsraten für Cephalosporine (65,1 %) bzw. Aminopenicillinen/BLI (74,4 %). Für die Kombination Vancomycin + Meropenem ergab sich mit 91,9 % die höchste hypothetische Sensibilität.

**Diskussion:**

Im Abgleich mit dem vorliegenden, einrichtungsspezifischen Keimspektrum erscheint die Kombinationstherapie Vancomycin + Meropenem für die empirische Therapie sinnvoll, sollte jedoch Patienten mit mehrfachen Revisionseingriffen oder septischen Infektionsverläufen vorbehalten bleiben, um die Selektion hochresistenter Keime zu vermeiden.

**Graphic abstract:**

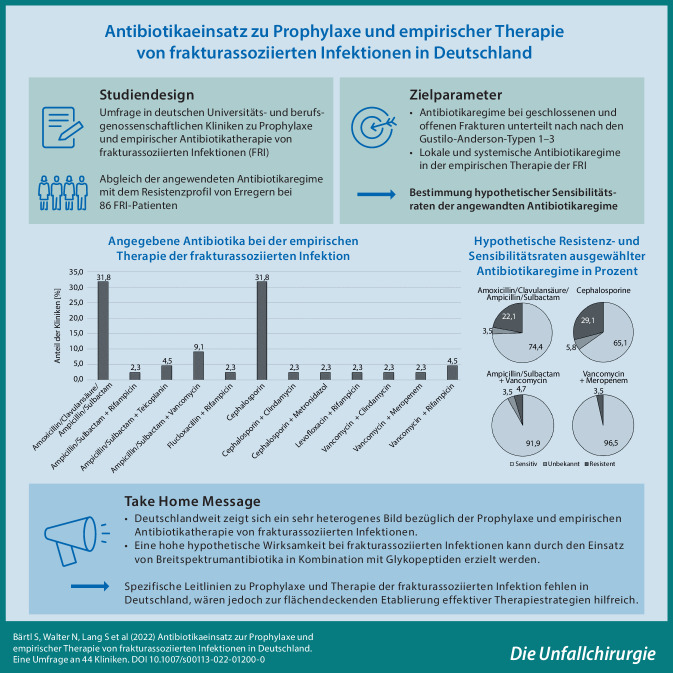

**Zusatzmaterial online:**

Die Online-Version dieses Beitrags (10.1007/s00113-022-01200-0) enthält zusätzlich den in der Umfrage verwendeten Fragebogen.

## Abstract (en)

**Background:**

Antibiotics play an important role in the prophylaxis and treatment of infections in orthopedic trauma surgery; however, there seems to be remarkable differences in the clinical antibiotic practice between hospitals, particularly for open fractures and for the empirical therapy of fracture-related infections (FRI).

**Methods:**

Therefore, we intended to evaluate the current clinical praxis in university and workers’ compensation hospitals in Germany with a questionnaire on prophylaxis and empirical treatment of FRI. The results were compared with the resistance profile of 86 FRI patients in order to analyze the hypothetical effectiveness of the empirical treatment.

**Results:**

A total of 44 hospitals (62.0%) responded. A homogeneous antibiotic prophylaxis (95.5% of all hospitals) with cephalosporins was reported for perioperative prophylaxis of internal fixation of closed fractures. For open fractures, eight different monotherapy and combination treatment concepts were reported. In empirical treatment of FRI, 12 different therapeutic concepts were reported, including aminopenicillins/beta lactamase inhibitors (BLI) (31.8%), cephalosporins (31.8%), and ampicillin/sulbactam + vancomycin (9.1%). In terms of the hypothetical effectiveness of these antibiotic regimes, low sensitivity rates of 65.1% and 74.4% for cephalosporins and aminopenicillins/BLI, respectively, were found. For the combination vancomycin + meropenem, the highest hypothetical sensitivity (91.9%) was detected.

**Discussion:**

Based on the existing, institution-specific pathogen spectrum, the combination therapy including meropenem and vancomycin seems to be of value but should be restricted to patients with multiple revision procedures or a septic course of infection in order to prevent the selection of highly resistant pathogens.

Frakturassoziierte Infektionen (FRI) bergen eine enorme Belastung für das Gesundheitssystem. Während in der perioperativen Antibiotikumprophylaxe bei geschlossenen Frakturen die einmalige Gabe von Cephalosporinen der 1. und 2. Generation etabliert ist, so scheint es in der Prophylaxe und empirischen Therapie von FRI große Unterschiede zu geben. In diesem Beitrag werden in einer deutschlandweiten Befragung prophylaktische und empirische Antibiotikaregime bei FRI erfasst und im Kontext publizierter Resistogramme von Erregern bei FRI-Patienten beleuchtet, um die hypothetische Wirksamkeit der jeweiligen Therapien zu ermitteln.

## Hintergrund und Fragestellung

Mit den Worten „Vom Ende einer qualvollen Therapie im Streckverband“ beschrieb Kuner [11] den revolutionären Wandel von der konservativen zur operativen Frakturbehandlung mit Einführung der Osteosynthese. Neben den zahlreichen Vorteilen dieser Verfahren birgt das Einbringen von Fremdmaterial das erhöhte Risiko einer frakturassoziierten Infektion (engl.: „fracture-related infection“, FRI) [5]. Die Infektionsraten können von 1–2 % bei geschlossenen Frakturen bis hin zu mehr als 30 % bei drittgradig offenen Frakturen nach Gustilo und Anderson (GA) reichen [12]. In Deutschland liegt die Inzidenz der FRI bei 11/100000 Einwohner [19] und stellt damit sowohl sozioökonomisch als auch für den einzelnen Patienten eine erhebliche Belastung dar [13, 18]. Somit hat die Prävention der FRI oberste Priorität. In Deutschland liegen zwar allgemeine Empfehlungen zur perioperativen Antibiotikumprophylaxe vor [1], einheitliche Leitlinien bezüglich der Auswahl innerhalb verschiedener Antibiotikaklassen existieren jedoch nicht. Die Paul-Ehrlich-Gesellschaft empfiehlt bei geschlossenen Frakturen die perioperative Anwendung von Cephalosporinen oder Aminopenicillinen/β-Lactamase-Inhibitor (BLI); bei höhergradigen offenen Frakturen sollen zudem Anaerobier erfasst werden [17]. Expertenempfehlungen befürworten auch den Einsatz lokaler Antibiotika (AB) sowohl bei der Infektionsprävention bei komplexen offenen Frakturen als auch in der Behandlung der FRI [6].

In Bezug auf die empirische Behandlung der FRI, d. h. zu einem Zeitpunkt zu dem der Erreger aus den intraoperativ gewonnenen mikrobiologischen Proben bzw. die entsprechenden Resistenzprofile noch nicht vorliegen, gibt es innerhalb Deutschlands keine spezifischen Empfehlungen. In einem kürzlich veröffentlichten Konsenspapier wird empfohlen, die empirische Therapie auf lokale Resistenzraten, die verfügbaren AB und patientenindividuelle Risikofaktoren abzustimmen. Die antibiotische Therapie sollte demnach Breitspektrum-AB, wie Lipopeptide oder Glykopeptide, enthalten und gramnegative Erreger erfassen [6]. Inwiefern diese Empfehlungen in Deutschland zur Anwendung kommen, bleibt unklar.

Ziele der vorliegenden Studie waren es deshalb, (1) die aktuellen lokalen und systemischen antibiotischen Therapiestandards in der Prophylaxe und Therapie der FRI an deutschen Kliniken zu erfassen und (2) diese im Kontext hypothetischer Resistenz- bzw. Sensibilitätsraten von Erregern bei FRI zu evaluieren, um daraus eine entsprechende Empfehlung zur AB-Therapie ableiten zu können.

## Methodik

### Umfrage zur Praxis der Antibiotikaanwendung

An deutschen Universitäts- und berufsgenossenschaftlichen Kliniken (BG-Kliniken), jeweils in den Fachbereichen Orthopädie und Unfallchirurgie, wurde eine Umfrage zur prophylaktischen Antibiotikagabe bei der Versorgung geschlossener bzw. offener Frakturen und zur empirischen Therapie von FRI durchgeführt. Im Januar 2021 wurden die Chefärzte bzw. Klinikdirektoren der Abteilungen für Orthopädie und Unfallchirurgie an insgesamt 71 Kliniken per E‑Mail kontaktiert und um Teilnahme an dieser Fragebogenaktion gebeten. Erinnerungen wurden 2‑mal im Abstand von 14 Tagen verschickt. Der versendete Fragebogen beinhaltete offene Fragen zu systemischer und lokaler Antibiotikaprophylaxe; hierbei wurde zwischen geschlossenen Frakturen und offenen Frakturen nach den GA-Typen 1–3 unterschieden sowie die im jeweiligen Haus praktizierte empirische AB-Therapie bei FRI erfragt (Zusatzmaterial online). Berücksichtigt wurden hierbei ausschließlich i.v.-AB-Therapien.

### Hypothetische Ermittlung der Wirksamkeit der empirischen Therapien

Um eine hypothetische Wirksamkeit der praktizierten empirischen AB-Therapien zu bestimmen, wurden die Umfrageergebnisse anschließend mit Resistenzprofilen von infektionsauslösenden Keimen von 86 FRI-Patienten aus dem eigenen Krankengut verglichen. Für diese Patienten wurde die mikrobiologische Datenbank nach Erregern durchsucht, welche durch matrixunterstützte Laser-Desorption/Ionisation (MALDI-TOF-MS) sowie durch antimikrobielle Empfindlichkeitstests nach den Richtlinien des *European Committee on Antimicrobial Susceptibility Testing* (EUCAST) nachgewiesen wurden [14].

### Statistische Auswertung

Die deskriptive und statistische Datenanalyse wurde mit der Software IBM SPSS Statistics durchgeführt (Version 24.0, Fa. IBM Corp, Armonk, NY, USA).

## Ergebnisse

Insgesamt nahmen 44 (9 BG-Kliniken und 35 Universitätskliniken) von insgesamt 71 angefragten Kliniken an der Umfrage teil, was einem Anteil von 62 % entspricht. Sämtliche der 44 Fragebogen konnten vollständig ausgewertet werden.

### Antibiotikaprophylaxe bei der operativen Versorgung von geschlossenen Frakturen

Es zeigte sich ein homogenes Bild bei der prophylaktischen Antibiotikagabe im Rahmen der Behandlung geschlossener Frakturen. Hier gaben 95,5 % der Kliniken an, Cephalosporine der 1. und 2. Generation zu verwenden (Tab. 1).

**Table 1:** 

Antibiotikum	Geschlossene Fraktur	Offene Fraktur		
		GA-Typ 1	GA-Typ 2	GA-Typ 3
Keine Angabe/keine Behandlung	2 (4,5 %)	7 (15,9 %)	7 (15,9 %)	7 (15,9 %)
Aminopenicilline/BLI	–	9 (20,5 %)	10 (22,8 %)	14 (31,8 %)
Piperacillin/Tazobactam	–	–	2 (4,5 %)	5 (11,4 %)
Meropenem	–	–	–	1 (2,3 %)
Cephalosporine (1. und 2. Generation)	42 (95,5 %)	26 (59,1 %)	20 (45,5 %)	11 (25,0 %)
Cephalosporin + Clindamycin	–	–	2 (4,5 %)	2 (4,5 %)
Cephalosporin + Gentamicin	–	–	–	1 (2,3 %)
Cephalosporin + Metronidazol	–	2 (4,5 %)	3 (6,8 %)	3 (6,8 %)

### Antibiotikaprophylaxe bei offenen Frakturen

Bei offenen Frakturen variierte die angewendete Antibiotikaprophylaxe erheblich (Tab. 1). Während bei GA-Typ‑1 und GA-Typ-2-Frakturen Cephalosporine der 1. und 2. Generation am häufigsten verwendet werden (59,1 % bei GA-Typ-1-Frakturen, 45,5 % bei GA-Typ-2-Frakturen), kommen häufiger auch Aminopenicilline/BLI zum Einsatz (20,5 % GA-Typ 1, 22,8 % GA-Typ 2). Bei GA-Typ-3-Frakturen werden sowohl Aminopenicilline/BLI (31,8 %), Cephalosporine der 1. und 2. Generation (25,0 %), Piperacillin/Tazobactam (11,4 %) sowie die Kombination aus Cephalosporinen der 1. und 2. Generation mit einem weiteren Antibiotikum (13,6 %) häufig eingesetzt.

### Empirische Antibiotikaregime für die Behandlung von FRI

**Figure 1:**
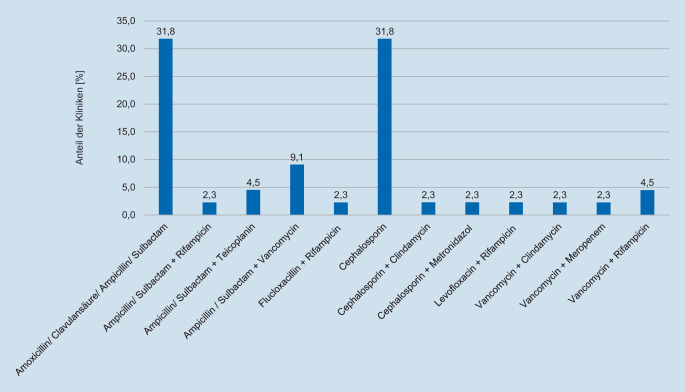

Bei der empirischen antibiotischen Behandlung zeichnete sich mit insgesamt 12 verschiedenen Mono- oder Kombinationstherapien ein inhomogenes Bild ab (Abb. 1). Am häufigsten wird eine Monotherapie mit einem Aminopenicillin/BLI verwendet (31,8 %), ebenso häufig Cephalosporine der 1. und 2. Generation (31,8 %). Darüber hinaus haben sich Kombinationstherapien etabliert, wobei die häufigste Kombination Aminopenicillin/BLI + Vancomycin ist (9,1 %).

**Figure 2:**
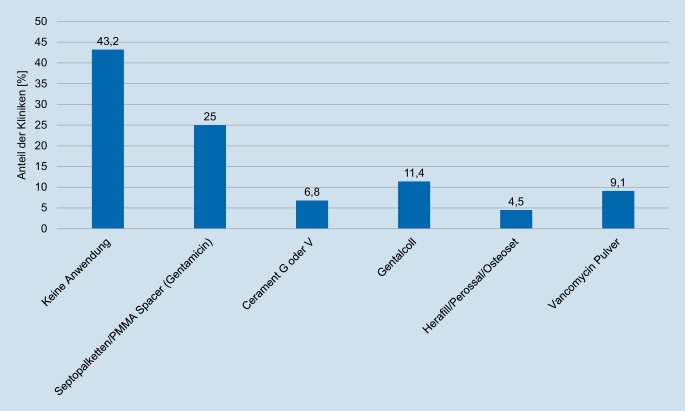

Ebenso uneinheitlich waren die Rückmeldungen zur lokalen Antibiotikaverwendung bei der Behandlung der FRI, wobei 43,2 % der Kliniken angaben, keine lokalen AB zu verwenden. Bei 56,8 % der Kliniken kommt am häufigsten gentamicinversetzter Knochenzement (25,0 %) in Form von Ketten oder Spacern zum Einsatz, gefolgt von gentamicinenthaltenden Kollagenschwämmen (GENTA-COLL®, Resorba Medical GmbH, Nürnberg, Deutschland) (11,4 %) (Abb. 2).

### Hypothetische Sensibilitäts- und Resistenzraten für die angewandten empirischen Antibiotikaregime

**Figure 3:**
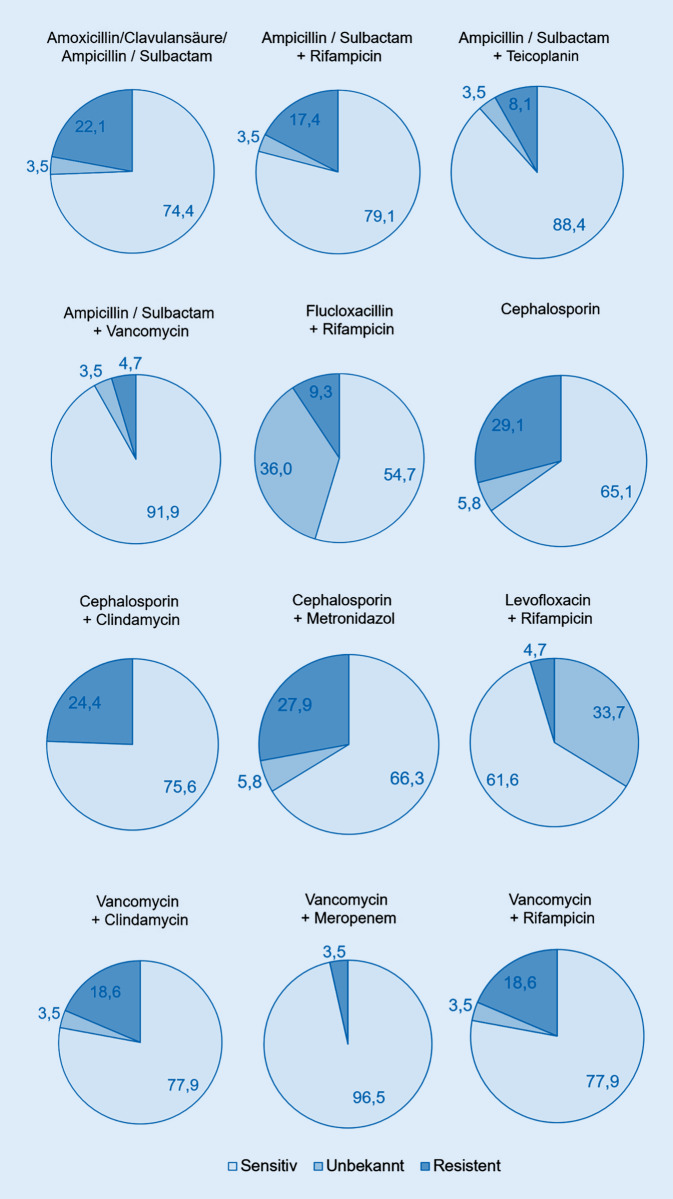

Die in der Umfrage angegebenen antimikrobiellen Therapieregime bei FRI wurden im Anschluss mit dem Erregerspektrum und den dazugehörigen Antibiogrammen einer zuvor analysierten Patientenkohorte mit FRI verglichen (*n* = 86) (Abb. 3; Tab. 2; [14]). Für die am häufigsten eingesetzten Monotherapien (Cephalosporine der 1. und 2. Generation oder Aminopenicilline/BLI) ergaben sich hierbei hypothetische Wirksamkeiten von 65,1 % bzw. 74,4 %. Diese ließen sich auch durch die Kombination von Cephalosporinen der 1. und 2. Generation mit Metronidazol oder Clindamycin lediglich auf 66,3 % bzw. 75,6 % steigern. Hingegen stellten sich Kombinationen von Aminopenicillinen/BLI mit Vancomycin oder Teicoplanin, die in 6 der untersuchten Krankenhäusern (13,6 %) als empirische AB-Therapie etabliert sind, mit einer hypothetischen Sensibilität von 91,9 % und 88,4 % als hochwirksam dar. Die höchste hypothetische Sensibilität (96,5 %) würde mit der Kombinationstherapie Meropenem + Vancomycin erreicht werden, welche von einer befragten Klinik als empirischer Therapiestandard genannt wurde.

**Table 2:** 

Antibiotikum	Umfrageergebnis	Hypothetische Sensibilität (%)
Aminopenicilline/BLI	14 (31,8 %)	74,4
Ampicillin/Sulbactam + Rifampicin	1 (2,3 %)	79,1
Ampicillin/Sulbactam + Teicoplanin	2 (4,5 %)	88,4
Ampicillin/Sulbactam + Vancomycin	4 (9,1 %)	91,9
Flucloxacillin + Rifampicin	1 (2,3 %)	54,7
Cephalosporine (1. und 2. Generation)	14 (31,8 %)	65,1
Cephalosporin + Clindamycin	1 (2,3 %)	75,6
Cephalosporin + Metronidazol	1 (2,3 %)	66,3
Levofloxacin + Rifampicin	1 (2,3 %)	33,7
Vancomycin + Clindamycin	1 (2,3 %)	77,9
Vancomycin + Meropenem	1 (2,3 %)	96,5
Vancomycin + Rifampicin	2 (4,5 %)	77,9

## Diskussion

Die vorliegende deutschlandweite Befragung ergab bezüglich der perioperativen Infektionsprävention bei geschlossenen Frakturen ein einheitliches Bild, während die Therapieschemata sowohl bei der Antibiotikaprophylaxe offener Frakturen als auch bei der empirischen systemischen und lokalen AB-Therapie von FRI eine erhebliche Heterogenität aufwiesen.

In Einheit mit den gegenwärtigen Leitlinien [1, 17] sind bei der operativen Versorgung von geschlossenen Frakturen in 95,5 % der befragten Kliniken Cephalosporine der 1. und 2. Generation als Therapiestandard etabliert. Zunehmend heterogen stellt sich dies bei offenen Frakturen dar. Während bei GA-Typ-1-Frakturen weiterhin Cephalosporine der 1. und 2. Generation eingesetzt werden, so kommen bei höhergradig offenen Frakturen häufiger Aminopenicilline/BLI, aber auch Breitband-AB wie Piperacillin/Tazobactam zum Einsatz. Die *Surgical Infection Society* empfiehlt lediglich die kurzzeitige Behandlung mit einem Cephalosporin der 1. Generation für alle GA-Typen [8], während andere Autoren für eine Abdeckung gramnegativer Erreger bei höhergradigen bzw. auch jeglichen offenen Frakturen plädieren [6, 7, 10]. Deutsche Leitlinien verweisen auf Empfehlungen der Paul-Ehrlich-Gesellschaft, in denen die Gabe von Aminopenicillinen/BLI oder Cephalosporinen und je nach Verschmutzungsgrad eine Abdeckung von gramnegativen Erregern empfohlen wird [1, 17]. So haben sich trotz des breiten Konsenses über den Nutzen der perioperativen Antibiotikaprophylaxe noch keine einheitlichen Therapiestandards etabliert, und entsprechend den diversen Empfehlungen variieren die angewendeten Therapieschemata.

Bei der empirischen Therapie der FRI wurden Cephalosporine der 1. und 2. Generation und Aminopenicilline/BLI gleichermaßen angegeben, wobei auch häufiger Kombinationstherapien zum Einsatz kommen. Insgesamt wurden 12 verschiedene Therapieschemata genannt. Nach Expertenempfehlungen soll die empirische Therapie auf lokale Resistenzen und patientenbezogene Faktoren abgestimmt werden, ein breites Spektrum, inklusive gramnegativer Erreger, abdecken und ein Lipopeptid oder Glykopeptid enthalten [4]. In Deutschland fehlen spezifische Empfehlungen für FRI und basieren auf Daten zu Endoprotheseninfektionen oder zur Osteomyelitis [3]. Substanzen wie Vancomycin und Carbapeneme sollen demnach aufgrund von Nebenwirkungen und Resistenzbildungen nicht standardmäßig in der empirischen Therapie eingesetzt werden [3].

Trotz der Empfehlungen für einen Einsatz hochwirksamer Kombinationstherapien [4] gaben in der vorliegenden Befragung die meisten Kliniken Monotherapien an, für die relativ hohe hypothetische Resistenzraten (Cephalosporine der 1. und 2. Generation 29,1 % Resistenz, Aminopenicilline/BLI 22,1 % Resistenz) zu verzeichnen waren. Eine gute potenzielle Sensibilität ließ sich für die Kombinationstherapie von Vancomycin mit Aminopenicillinen/BLI oder Meropenem erreichen. Der zusätzliche Einsatz von Meropenem kann bei zunehmender Relevanz gramnegativer Erreger bei der FRI einen Vorteil bieten, sollte jedoch, bei nur geringfügiger Unterlegenheit gegenüber der Kombinationstherapie Aminopenicillinen/BLI + Vancomycin, Patienten mit mehrfachen Revisionseingriffen oder septischen Infektionsverläufen als Teil einer Last-Line-Behandlungsstrategie vorbehalten bleiben, um das Auftreten potenzieller Resistenzen gegenüber den genannten Reserveantibiotika zu vermeiden [15]. Der Nutzen von Vancomycin ist zudem in Deutschland aufgrund von niedrigen Raten von Methicillin-resistenten Staphylokokken und des Risikos der Nephrotoxizität zu diskutieren [2]. Zur Vermeidung von Nebenwirkungen ist hierbei ein therapeutisches Drugmonitoring im Sinne von Talspiegelbestimmungen dringend erforderlich [16]. Einheitliche Empfehlungen für die empirische Therapie der FRI existieren jedoch nicht, da die Wahl der geeigneten Substanzen von vielfältigen Faktoren, wie lokalen Resistenzraten und patientenindividuellen Faktoren, beeinflusst werden [4, 6]. Zudem muss der Nutzen einer frühzeitigen, empirischen AB-Therapie bei FRI-Patienten in weiteren Studien belegt werden [9].

Die lokale Antibiotikaanwendung wird inzwischen in der Prophylaxe und Therapie von FRI empfohlen, obgleich die Effektivität lokaler Antibiotika in weiteren klinischen Studien belegt werden muss [4, 6]. Hohe lokale Wirkspiegel, geringere systemische Nebenwirkungen und neue Trägermaterialen bieten einen vielversprechenden Ansatz [6]. Interessanterweise gaben 43,2 % der Kliniken an, keine lokalen AB zu verwenden, während die übrigen eine Vielzahl unterschiedlicher gentamicin- oder vancomycinbeladener Träger nannten. Gegenwärtig zeigt sich die Studienlage insbesondere bezüglich der Überlegenheit einzelner Materialen noch nicht ausreichend und erfordert weitere Untersuchungen [4, 6].

Die vorliegende Studie weist einige Limitationen auf: Die Auswahl der befragten Kliniken fokussierte sich auf deutsche Universitäts- und BG-Kliniken. Insbesondere an diesen Kliniken wurden eine große Erfahrung und Expertise in der Behandlung solcher Infektionen erwartet, jedoch kann die Umfrage nicht die Therapien im gesamten Krankenhaussektor in Deutschland repräsentieren. Gründe für diese Auswahl lagen in der Erwartung eines hohen wissenschaftlichen Interesses der eingeladenen Kliniken und der damit einhergehenden hohen Rücklaufquote. Außerdem wurde angenommen, dass insbesondere in diesen Kliniken ein multidisziplinärer Behandlungsansatz mit den Abteilungen für Infektiologie und Mikrobiologie gepflegt wird und dies zu einem homogeneren Therapiebild führen würde. Hierbei ist jedoch anzumerken, dass die hier vorliegende Heterogenität auch durch die im Rahmen von etablierten Antibiotic-Stewardship-Programmen exaktere Kenntnis des lokalen Keimspektrums am jeweiligen Standort und folglich spezifisch und lokal ausgerichtete empirische Antibiotikaregime begründet sein kann. Zudem lag der Fokus dieser Arbeit nicht auf der Dauer der prophylaktischen und empirischen AB-Therapie sowie auf der Berücksichtigung interindividueller Risikofaktoren, da die genannten Aspekte gegenwärtig immer noch sehr kontrovers diskutiert werden und deshalb weiterer prospektiver Studien bedürfen [4, 6]. Inwiefern die in dieser Untersuchung herausgestellte Heterogenität auch durch die Berücksichtigung spezifischer Risikofaktoren in Bezug auf den Patienten, den Traumamechanismus oder die Wundkontamination begründet ist, kann mit der vorliegenden Befragung nicht ausreichend beantwortet werden. Eine weitere Limitation besteht hinsichtlich der beschriebenen hypothetischen Wirksamkeit der empirischen Therapie beim Vorliegen einer FRI, da hier mit 86 FRI-Patienten ein eigenes Krankengut beschrieben ist, das nicht unbedingt einer repräsentativen Auswahl für alle Kliniken entsprechen muss. Daher sollte jede Klinik für sich selbst regelmäßig Resistenzprofile bestimmen und diese mit den praktizierten AB-Regimen abstimmen.

## Ausblick

Sowohl für die Infektionsprophylaxe bei offenen Frakturen als auch bei der empirischen Verwendung lokaler und systemischer AB zeigt sich an den befragten deutschen Klinken ein sehr uneinheitliches Bild. Häufig eingesetzte AB weisen erhebliche Resistenzraten bezüglich des Erregerspektrums bei FRI auf, während der Einsatz von lokalen AB an vielen Klinken noch nicht etabliert ist. Insbesondere bei schweren und komplexen Verläufen kann eine Kombination aus einem Breitspektrum-β-Lactam-Antibiotikum mit einem Glykopeptid sinnvoll sein. Weitere, prospektive Studien zu Wirksamkeit und Sicherheit dieser Kombinationstherapie könnten dazu beitragen, sichere und effektive Therapieempfehlungen zur empirischen AB-Therapie bei FRI zu formulieren.

## Fazit für die Praxis

- Deutschlandweit zeigt sich ein sehr heterogenes Bild bezüglich der Prophylaxe und empirischen Antibiotikumtherapie von frakturassoziierten Infektionen.
- Eine hohe hypothetische Wirksamkeit bei frakturassoziierten Infektionen kann durch den Einsatz von Breitspektrum-Antibiotika in Kombination mit Glykopeptiden erzielt werden.
- Der lokale Antibiotikaeinsatz hat sich in Deutschland noch nicht flächendeckend etabliert, bietet jedoch einen vielversprechenden Ansatz in der Behandlung von Knochen- und Gelenkinfektionen.
- Spezifische Leitlinien zu Prophylaxe und Therapie der frakturassoziierten Infektion fehlen in Deutschland, wären jedoch zur flächendeckenden Etablierung effektiver Therapiestrategien hilfreich.

## Supplementary Information




