# Supplementary material for: Antibiotic use for prophylaxis and empirical therapy of fracture-related infections in Germany: A survey of 44 hospitals
Source: Unfallchirurgie (Heidelb). 2022 Jun 24;126(9):707–14. [Article in German] doi: 10.1007/s00113-022-01200-0 (PMC10450009; doi:10.1007/s00113-022-01200-0)
Supplement: Supplementary file 1 [file 113_2022_1200_MOESM1_ESM.pdf]

Fragebogen – Antibiotische Infektprophylaxe und -therapie in Orthopädie und Unfallchirurgie

1. Systemische Antibiotikaprophylaxe

Welches **systemische** Antibiotikum wählen Sie in Ihrer Klinik standardmäßig zur Infektprophylaxe und wie lange verwenden Sie es bei

1.1 Standardosteosynthese (geschlossene Fraktur):

1.2 Offener Fraktur

1.2.1 Bei offener Fraktur Gustilo-Anderson Typ I

1.2.2 Bei offener Fraktur Gustilo-Anderson Typ II

1.2.3 Bei offener Fraktur Gustilo-Anderson Typ III

2. Lokale Antibiotika

2.1 Bei welchen offenen Frakturen benutzen sie lokale Antibiotikaträgern?

2.2 Welches Produkt benutzen Sie in diesem Fall?

3. Empirische systemische Antibiotikatherapie

Welches Antibiotikum oder welche Antibiotikakombination wählen Sie als empirische Therapie im Rahmen der Behandlung einer frakturassoziierten Implantatinfektion und in welcher Standarddosierung? (Operation bis zum Vorliegen der Resistogramme im Falle eines dann positiven Keimnachweises)
